# What Important Information Does Transesophageal Echocardiography Provide When Performed before Transvenous Lead Extraction?

**DOI:** 10.3390/jcm13175278

**Published:** 2024-09-05

**Authors:** Dorota Nowosielecka, Wojciech Jacheć, Małgorzata Stefańczyk Dzida, Anna Polewczyk, Dominika Mościcka, Agnieszka Nowosielecka, Andrzej Kutarski

**Affiliations:** 1 Department of Cardiac Surgery, The Pope John Paul II Province Hospital, 22-400 Zamość, Poland; 2 Department of Cardiology, The Pope John Paul II Province Hospital, 22-400 Zamość, Poland; 3 2nd Department of Cardiology, Faculty of Medical Sciences in Zabrze, Medical University of Silesia in Katowice, 41-800 Zabrze, Poland; wjachec@interia.pl; 4 Department of Physiology, Pathopysiology and Clinical Immunology, Collegium Medicum of Jan Kochanowski University, 25-369 Kielce, Poland; annapolewczyk@wp.pl; 5 Department of Cardiac Surgery, Swietokrzyskie Cardiology Center, 25-736 Kielce, Poland; 6 Internal Medicine Residency Program, Tallahassee Memorial HealthCare, Florida State University, 1300 Miccosukee Road Tallahassee, Tallahassee, FL 32308, USA; dominikamoscicka@gmail.com; 7 Department of Internal Medicine and Geriatrics, The A. Falkiewicz Specialist Hospital, 52-114 Wrocław, Poland; ag.nowosielecka@gmail.com; 8 Department of Cardiology, Medical University, 20-059 Lublin, Poland; a_kutarski@yahoo.com

**Keywords:** transesophageal echocardiography, transvenous lead extraction, scar tissue, vegetation, perforation, lead loop, major complications, mortality

## Abstract

**Background:** Transesophageal echocardiography (TEE) is mandatory before transvenous lead extraction (TLE), but its usefulness remains underestimated. This study aims to describe the broad range of TEE findings in TLE candidates, as well as their influence on procedure complexity, major complications (MCs) and long-term survival. **Methods:** Preoperative TEE was performed in 1191 patients undergoing TLE. **Results:** Lead thickening (OR = 1.536; *p* = 0.007), lead adhesion to heart structures (OR = 2.531; *p* < 0.001) and abnormally long lead loops (OR = 1.632; *p* = 0.006) increased the complexity of TLE. Vegetation-like masses on the lead (OR = 4.080; *p* = 0.44), lead thickening (OR = 2.389; *p* = 0.049) and lead adhesion to heart structures (OR = 6.341; *p* < 0.001) increased the rate of MCs. The presence of vegetations (HR = 7.254; *p* < 0.001) was the strongest predictor of death during a 1-year follow-up period. **Conclusions:** TEE before TLE provides a lot of important information for the operator. Apart from the visualization of possible vegetations, it can also detect various forms of lead-related scar tissue. Build-up of scar tissue and the presence of long lead loops are associated with increased complexity of the procedure and risk of MCs. Preoperative TEE performed outside the operating room may have an impact on the clinical decision-making process, such as transferring potentially more difficult patients to a more experienced center or having the procedure performed by the most experienced operator. Moreover, the presence of masses or vegetations on the leads significantly increases 1-year and all-cause mortality.

## 1. Introduction

Transthoracic echocardiography (TTE) and transesophageal echocardiography (TEE) are well-established as techniques for the assessment of cardiac implantable electronic devices (CIEDs), especially in patients with lead-related complications [1,2,3,4,5,6,7,8,9]. TEE is particularly useful in the identification of infectious complications and essential in diagnosing infective endocarditis [5,6,7,8,9,10]. Recently, TEE has also been used to monitor transvenous lead extraction (TLE) procedures [2,3,11,12,13,14]. In the last few decades, transvenous lead extraction has been regarded as a first-line strategy in the management of lead-related complications [1,2,3]. The guidelines on lead extraction recommend the use of transesophageal or intracardiac echocardiography as a basic and mandatory monitor to improve the safety of the procedure [2,3,11,12,13,14]. According to various reports, the risk of major complications and death during TLE ranges from 0.4 to 3.4% and from 0.0 to 0.5%, respectively [15,16,17,18,19,20].

Apart from intraoperative monitoring, the echocardiographic assessment before and after the procedure is likewise important. Currently, TEE is a widely available method, possible with not only 2D imaging but also with a 3D and a 4D option, recorded in real time [11,12,13,14,21,22,23,24]. Therefore, it allows for a very precise visualization of the intracardiac route of the lead(s) and the consequences of their placement inside heart chambers.

The echocardiography protocol in patients with implanted devices differs from the standard one, and additional evaluation is required, especially in patients prior to TLE. Particular attention should be paid to the consequences of the presence of leads inside the heart, which may potentially affect the course of the future TLE procedure [11,12,13,25]. The information obtained from the preoperative examination aids clinicians in predicting procedure complexity and possible complications related to lead extraction. Therefore, TEE is a valuable supplement to fluoroscopy when choosing the right TLE strategy. The study explores the consequences of long lead implant duration manifesting as different forms and locations of scar tissue build-up, as well as other lead-related findings which may influence the strategy of TLE and further therapeutic decisions.

## 2. Methods

### 2.1. Study Population

Of the 1322 consecutive patients undergoing TLE between June 2015 and March 2022, 1191 had a preoperative TEE. The remaining 131 patients in whom TEE examination was not performed for medical contraindications to esophageal tube insertion (26 patients with resistance to tube insertion, esophageal diverticula, varices, bleeding, etc.), or due to incomplete description of the findings or incomplete clinical data (105 patients), were excluded from the study. All information relating to patients, TEE and procedures was entered into the computer on an ongoing basis.

### 2.2. Lead Extraction Procedure

TLE was defined according to the guidelines on the management of lead-related complications (HRS 2009 and 2017, and EHRA 2018 [1,2,3]. All procedures were performed using mechanical systems (polypropylene Byrd dilator sheaths, Cook^®^ Medical, Leechburg, PA, USA), mainly via the implant vein. If technical difficulties arose, a different vascular access and/or additional tools were used. Laser energy was not applied [17,26]. TLE was performed in patients under general anesthesia with preparation of the surgical field for cardiac surgery. The radial artery was used for continuous invasive blood pressure monitoring. The team consisted of an operator having experience with pacing therapy, a second cardiologist, a cardiac surgeon, an anesthesiologist and a cardiologist with experience in TEE [26].

Non-infectious TLE indications included lead replacement (mechanical lead damage), lead dysfunction, upgrades, abandoned leads/prevention of abandonment, threatening/potentially threatening leads (loops, free ends, left heart, lead-related tricuspid valve regurgitation), the re-establishing of venous access and other indications (MRI indications, cancer, painful pockets or loss of indications for pacing).

### 2.3. Preoperative TEE

TEE monitoring during the TLE procedure is the current standard [1,2,3]. In practice, we perform a preoperative TEE examination in every patient without contraindications to the insertion of the TEE probe into the esophagus. The preoperative examination is performed when the patient is already under general anesthesia, while the surgical team prepares the patient and the operating field for TLE.

In all patients, preoperative TEE was performed using multi-plane probes 6VT-D (General Electric Company, Boston, MA, USA) or X7-2t Live 3D (Phillips Healthcare, Andover, MA, USA), and the findings were documented in the medical records of each patient. The recorded echocardiograms were retrospectively reassessed for diagnostic accuracy. Leads were assessed in standard esophageal and transgastric views, and, if necessary, non-standard imaging planes were used for better visualization of the structures [11,12,13,24,25]. The examination began after intubation to assess lead position, extent of fibrous encapsulation, lead-to-lead adhesions and additional structures on the leads, as well as function of the tricuspid valve (TV) and the pericardium [25,26,27].

### 2.4. Definitions of Additional Structures on the Leads and Other Echocardiographic Findings

**Figure 1:**
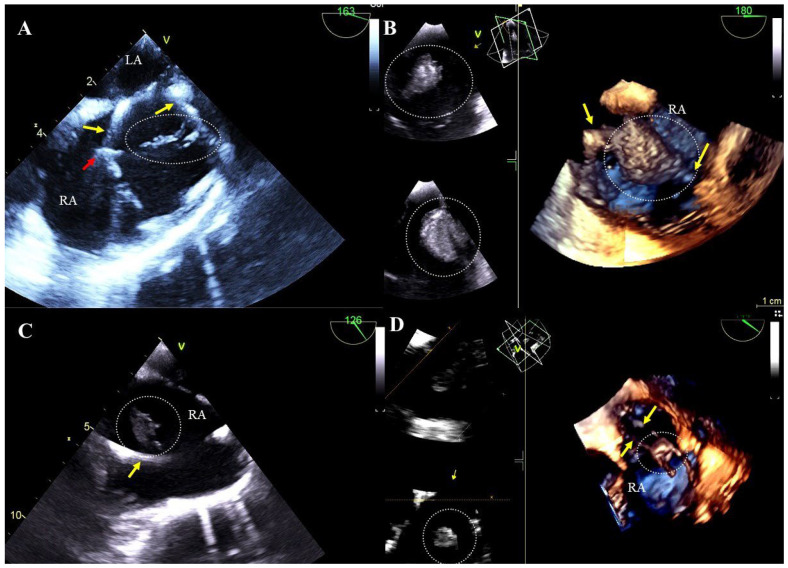
TEE (2D, 3D) before TLE showing additional structures on the leads. Mobile structures correspond to scar tissue on the atrial lead (dashed line) (**A**). The mass on the lead corresponds to a large thrombus visualized in the right atrium (**B**). In the right atrium, in the patient with no signs of infection, an additional structure on the lead was visualized, which may correspond to a veg-like structure (**C**). In the right atrium, in the patient with no signs of infection, an additional structure was binding two leads; this may represent scar tissue or a veg-like structure (**D**). (Yellow arrows mark the electrodes, and additional structures are displayed in circles.).

Floating scar tissue attached to the lead: small, hyperechoic structures with uneven contours, more rigid and less mobile than clots of uniform echogenicity being components of scar tissue developed in response to lead presence. They probably arise from progressive fibrosis of non-lysed blood clots (Figure 1A, Movie S1).

Blood clots on the leads: most often airy, hypoechoic, flaccid, flag-like formations, sometimes large and hyperechoic with a smooth surface (Figure 1B, Movie S2).

Vegetation-like masses: well-saturated formations resembling vegetations in shape and mobility, associated with leads and recorded in patients without symptoms of infection. They are probably a residue of past infections (old fibrosed vegetations). It cannot be ruled out that these masses are unusual-looking old clots or adhesions (Figure 1C,D, Movie S3).

**Figure 2:**
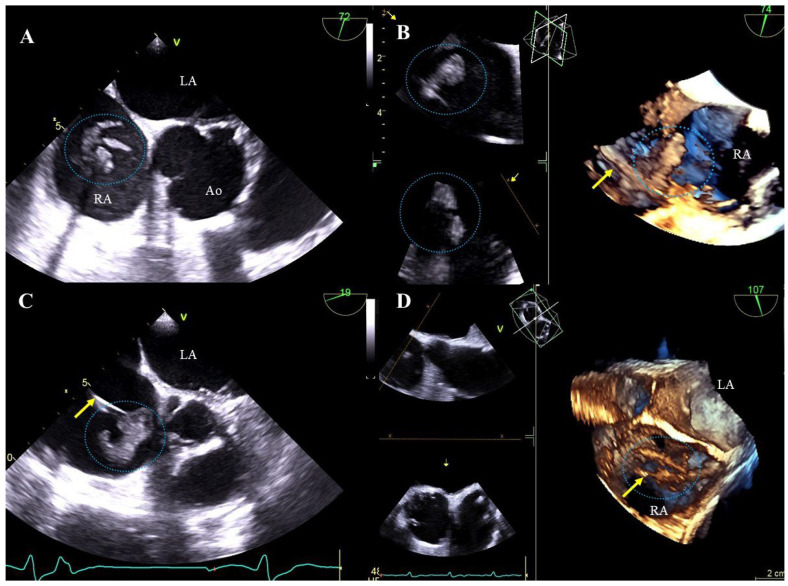
TEE images (2D, 3D) showing vegetations on CIED leads. Various-shaped structures representing bacterial vegetations (blue circles) are visualized on the leads (yellow arrow) in the right atrium. D TEE (**A**,**C**), 3D TEE (**B**,**D**).

Bacterial vegetations: multi-shaped, irregular, of varying size, balloting formations of heterogeneous echogenicity. Vegetations are diagnosed if there are signs of a generalized infection (positive inflammation markers, blood cultures) or local infection (generator pocket infection) (Figure 2A–D, Movie S4).

**Figure 3:**
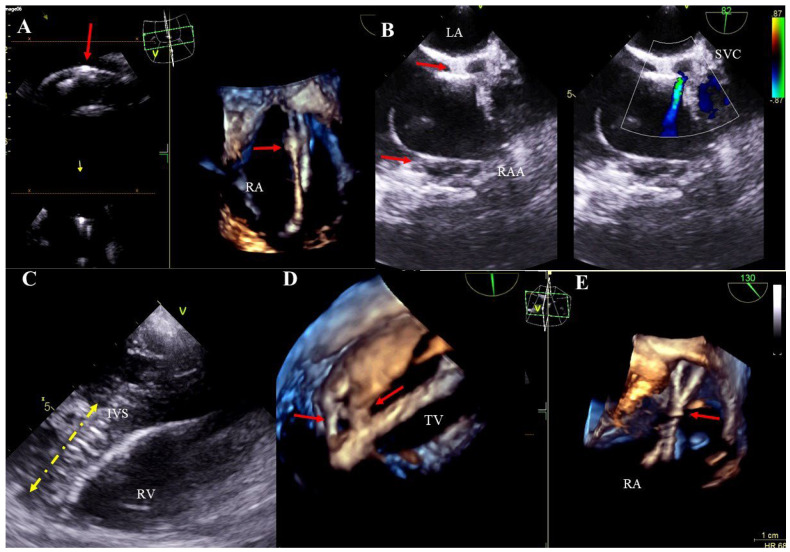
TEE (2D, 3D) showing scar tissue around the leads. Segmental thickening of the leads and lead-on-lead adhesions in the right atrium (red arrow) (**A**). Pathological attachment of the two leads to the interatrial septum and to the atrial wall near the atrial appendage (red arrows). The narrowing of the vena cava at entry into the atrium is caused by the thickened leads and pathological scar tissue (Doppler color) (**B**). Thickened ventricular lead (yellow line) pathologically attaches to the endocardium of the interventricular septum in the right ventricle (**C**). The image from the right ventricle depicts a pathological adhesion (red arrows) of the lead to the edge of the tricuspid valve leaflet (**D**). Binding and intersection of thickened leads in the atrium (red arrow) (**E**).

Lead thickening: segmental thickening and hyperechogenicity caused by the presence of scar tissue. Additionally, uneven outlines of the thickened lead segment with small mobile structures and with a consistent clinical picture may suggest an inflammatory process (Figure 3A–E, Movie S5).

Lead adhesion to surrounding structures: due to the presence of fibrous encapsulation, the lead adheres to the anatomical structures of the heart or the vein wall (Figure 3B–D, Movie S6). A characteristic feature is the joint movement of the bound elements. The term also includes lead-on-lead adhesions (two or three leads) that move together (Figure 3A,E, Movie S5). Attached masses binding the lead to the surrounding structures (vein wall or heart structures) are remnants of a previous asymptomatic inflammatory response to the presence of the lead in the heart. Over time, they may calcify or mineralize, crystallize or even ossify.

**Figure 4:**
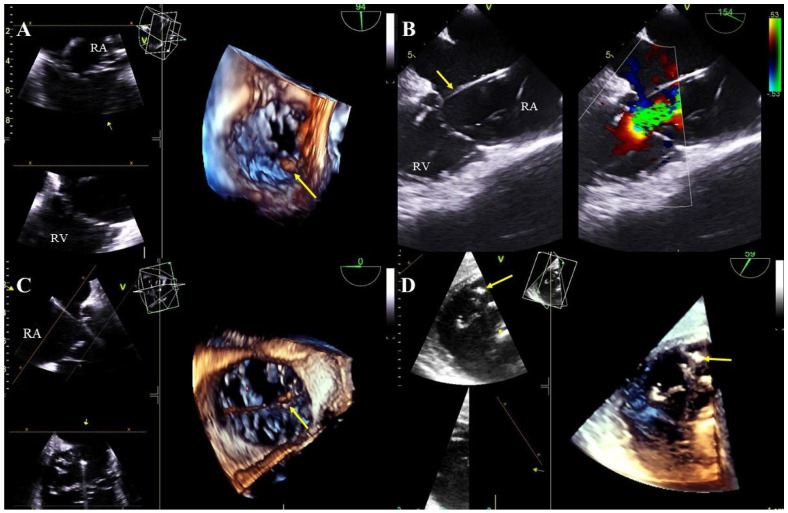
Tricuspid valve dysfunction caused by the presence of the electrode (TEE 2D, 3D). The lead (yellow arrow) in the tricuspid valve supports the septal leaflet and hinders proper coaptation of the leaflets (TEE 3D) (**A**,**C**). Severe tricuspid valve regurgitation resulting from the septal leaflet being pathologically supported by the lead (yellow arrow), (2D, color Doppler image from panel A) (**B**). The posterior leaflet of the tricuspid valve is perforated by the lead (yellow arrow) (**D**).

Lead-dependent tricuspid valve dysfunction (LDTVD): new valve regurgitation caused by the lead. The most common mechanism is impingement of the valve, adhesion of the lead to the leaflet or its perforation during implantation (Figure 4A–D, Movie S7).

**Figure 5:**
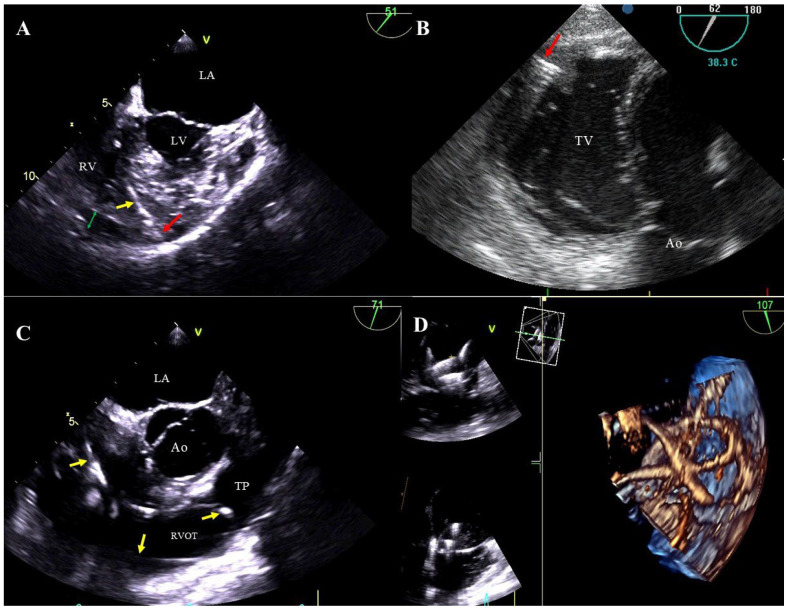
TEE (2D, 3D) showing perforations of the heart wall and excess lead loops. The ventricular lead (yellow arrow) perforating the wall of the right ventricle near the apex, visible in the pericardium (red arrow). Separation of pericardial layers—fluid accumulation (green arrow) (**A**). Perforation of the anterior wall of the right ventricle caused by the lead (red arrow) (transgastric view) (**B**). A long loop of the left ventricular lead (yellow arrows) dislodging to the pulmonary trunk (**C**). In the right atrium, tangled loops of two ventricular leads further impair the tricuspid valve function (**D**).

Lead-related cardiac perforation outside the pericardial sac: visualization of the tip of the lead beyond the outline of the heart wall in the pericardial space, often with pericardial effusion (Figure 5A,B, Movie S8).

Penetration of the heart wall with the lead: the location of the tip of the lead deep at the interface between the muscle and the pericardium. Usually asymptomatic and only as an echocardiographic finding (Movie S9).

Excessively long lead loops: excess length of the lead in the heart. Proper lead arcs should not touch the heart wall, especially the TV leaflets, due to the potential risk of adhesion to these structures and their subsequent dysfunction (Figure 5C,D, Movie S10).

### 2.5. Dataset and Statistical Methods

#### 2.5.1. Creation of Subgroups for Future Analysis

All patients were divided into 3 subgroups: (1) TLE for non-infectious indications: 930 patients; (2) TLE for isolated pocket infection: 63 patients, and (3) TLE for lead-related systemic infection (sepsis or bacteremia with or without vegetations) with coexisting pocket infection or not: 198 patients.

#### 2.5.2. Statistical Analysis

Continuous variables are presented as the mean ± standard deviation. The categorical variables are presented as numbers and percentages. The significance of differences between groups (1, 2, 3) was determined using the Chi^2^ test with Yates correction (Yates correction was applied when at least one of the frequencies observed of less than 10 was found) or the unpaired Mann-Whitney U test, as appropriate. The non-parametric Mann-Whitney U test was used due to unequal sizes of the samples and nonlinearity of most of the continuous variable distribution. Including the Bonferoni correction, *p* < 0.017 was considered statistically significant. To determine the significance of the impact of TEE findings on TLE complexity and major complications, univariable and multivariable logistic regression was used. Univariable and multivariable Cox regressions were used to determine the value of TEE findings as prognostic factors for death after TLE during the 1-year follow-up. *p* < 0.05 was considered statistically significant. Statistical analysis was performed using Statistica 13.3 (TIBCO Software Inc., Tulsa, OK, USA).

#### 2.5.3. Procedure Complexity

Procedure Complexity was measured using The Complex Indicator of the Difficulty of the TLE, which includes extraction time of all leads >20 min (2 points), average extraction time of a single lead >12 min (2 points) and the need to use a metal sheath or Evolution/TightRail, venous access other than lead implant vein or the need to use lasso catheters or basket catheters (each for 1 point). The sum of the points was the CID-TLE value [28].

#### 2.5.4. Approval of the Bioethics Committee

All patients gave their informed written consent to undergo TLE and use anonymous data from their medical records, approved by the Bioethics Committee at the Regional Chamber of Physicians in Lublin no. 288/2018/KB/VII. The study was conducted according to the ethical guidelines of the Declaration of Helsinki.

## 3. Results

Table 1 summarizes the basic characteristics of the study groups. TEE before TLE was performed in 1191 patients (females 38.96%), mean age 67.12 ± 14.74 years. Ischemic heart disease was the most common co-morbidity (64.40%). Patients with systemic infection were more likely to have more co-morbidities and, therefore, higher values of the Charlson index (6.35) compared to the non- infectious patients. Among the parameters derived from TTE before the procedure, compared to the non-infectious group, patients with systemic infection had lower LVEF and higher LVEDD and SPAP. Patients with a local pocket infection presented a lower NYHA functional class.

The following TEE findings were analyzed: LDTVD, various forms of scar tissue around the leads and the consequences of its occurrence, such as adhesions at all levels of the cardiovascular system, additional masses associated with the leads, excessively long lead loops in heart chambers, perforation of the heart wall by the lead and the presence of fluid in the pericardial cavity (Table 2).

In the entire group, LDTVD was detected in 6.72% of patients and adhesion of leads to the anatomical heart structures in 29.34%, whereas lead thickening was detected in 36.52%, with no significant differences between infectious and non-infectious subjects. Different forms of scar tissue were observed more frequently in the non-infectious group (40.11%). Bacterial vegetations were much more often detected via TEE than via TTE (77.78% vs. 28.06%), multiple vegetations were most common (56.06%) and the vast majority of them were associated with the leads 98.03%.

Additional structures on the lead included blood clots, vegetation-like masses and adhesions. Adhesions and blood clots were more likely in non-infectious patients (20.43% and 10.97%), whereas vegetation-like masses were more likely in patients with systemic infection (15.15%). Excessively long lead loops were observed at a comparable rate in all groups (19.40%). Perforation was reported in 10.16% of the study population and significantly more often in the non-infectious group (11.82%). Penetration was less frequent (5.54%). Fluid in the pericardial cavity was found in 7.89% of all patients and in as many as 10.61% of patients with endocarditis.

Table 3 summarizes TEE findings and their impact on procedure complexity, complications and long-term survival. Different forms of scar tissue on the leads (74.22 vs. 53.98%; *p* < 0.001) such as lead thickening (47.30 vs. 33.19%; *p* < 0.001), lead adhesions at different levels of the cardiovascular system (47.27 vs. 23.72%; *p* <0.001), lead-on-lead adhesion (43.20 vs. 25.22%; *p* < 0.001) and lead loops in the heart (27.18 vs. 16.93%; *p* < 0.001) were more often associated with increased TLE complexity, whereas vegetations were less frequent (8.36 vs. 14.38%; *p* = 0.011).

Multivariable regression analysis showed that lead thickening (OR = 1.536; *p* = 0.007), lead adhesion to the anatomical heart structures (OR = 2.531; *p* < 0.001) and lead loops in the heart (OR = 1.632; *p* = 0.006) were independent predictors of increased procedure complexity.

Any form of scar tissue on the leads (93.10 vs. 58.00%; *p* < 0.001)—such as vegetation-like masses (6.90 vs. 3.36%), adhesions (41.38 vs. 17.21%; *p* = 0.002), lead adhesions at different levels of the cardiovascular system (82.14 vs. 28.03%; *p* <0.001) and lead-on-lead adhesion (82.75 vs. 28.23%; *p* < 0.001)—were more common in patients with major complications. Multivariable regression analysis demonstrated that vegetation-like masses on the lead (OR = 4.080; *p* = 0.44), lead thickening (OR = 2.389; *p* = 0.049) and lead adhesion to the heart structures (OR = 6.341; *p* < 0.001) tended to increase the rate of major complications.

## 4. Discussion

Performing a TEE before TLE is helpful for detecting lead-related changes in the heart [5,7,9,10,11,12,13,21,22,23,24,25,26,27]. The information obtained from the examination performed by an experienced echocardiographer allows the extractor to predict the level of procedure complexity and its major complications [25,26]. It also helps in a better planning of the lead extraction strategy and preparation of the team to take immediate action in the event of acute cardiac tamponade, the most dangerous complication of lead extraction.

The presence of mobile masses attached to the leads in asymptomatic patients is a well-known phenomenon, whereas data on the frequency of their occurrence are ambiguous, dependent on the diagnostic method and the study population. In TTE, additional structures on the leads are visible only in a small proportion of patients (approx. 1.6%) [29]. In TEE, the proportion of such individuals increases to 28% [5,8,9,10], and, in ICE, random structures on the leads are found in 30% [6,7,9,30]. It has not been shown that asymptomatic, mobile masses on the leads have a significant impact on the course and safety of the procedure.

Many investigators have also confirmed the adverse effects of lead-related endocarditis on the life span of patients [31,32,33,34,35,36].

Small, multiple masses attached to the leads are the most common type of vegetations. Their presence does not significantly affect the course of the procedure. The occurrence of large vegetations >2–3 cm is currently less common; in this study, large vegetations were detected in only 2.68% of all infections. If they occur, special attention should be paid to their mobility, size and potential risk of migration during lead removal. In such a situation, pulmonary protection devices should be used during the procedure or the transvenous approach should be abandoned (based on the authors’ personal experience). These measures should be taken to avoid the risk of massive septic pulmonary embolism [1,4,14,24].

The occurrence of lead-associated structures resembling vegetations in candidates for TLE for reasons other than infection deserves attention. Vegetation-like masses were rare in the entire population (3.44%). In the infection group they were detected in 15.15%. Patients were assigned to groups based on retrospective evaluation of the data (medical history and biochemical and bacteriological test results) and online measurements from real time echocardiography, without retrospective verification. Presumably, vegetation-like structures are remnants of old vegetations in asymptomatic patients or an underdiagnosed disorder (based on the authors’ personal observations). It should be remembered that in echocardiographic examinations there are still no certain and unambiguous characteristics that would allow us to clearly distinguish between the infectious and non-infectious origin of the imaged structures. In doubtful situations, additional diagnostic methods should be used, such as 18-fluorodeoxyglucose PET/computed tomography (FDG-PET/CT) and ^99m^Tc labelled hexamethylopropylene amine oxime white blood cell scintigraphy (WBC SPECT) [14].

Apart from mobile structures, there are also stationary masses associated with leads, which are so far not widely presented in the available literature. They are formed by a layer of scar tissue surrounding the lead, causing its thickening, which is characterized by increased echogenicity in ultrasound imaging [10,26,27].

Immediately after CIED implantation, as a result of vascular or endocardial irritation, a clot forms on the lead which, if not degraded, transforms into fibrous encapsulation. A fibrous envelope binds the leads to the structures of the heart and other leads. Build-up can occur at any level of the cardiovascular system, often on multiple levels in a single patient (40.10%). The most common finding was lead adhesion to the right ventricular endocardium and the tricuspid apparatus. Fibrous tissue is a “silent actor” during the extraction procedure. Other investigators have also confirmed the role of fibrous tissue in the assessment of TLE complexity [37,38].

However, no one has presented definite evidence that it is one of the main factors which significantly increases the risk of major complications and the level of procedure difficulty. Paradoxically, it may have a protective effect on the short- and long-term survival of patients, which was demonstrated in our previous study [26] and partially confirmed in the present investigation. Scar tissue is formed in response to the presence of a foreign body, such as the lead. Younger patients, without additional diseases and with increased life expectancy, show a faster and more intense defensive reaction and formation of scar tissue. Predicted survival of these patients is therefore longer, regardless of the TLE procedure they have undergone, and this should explain the relationship [26].

Excessive lead loops is another echocardiographic finding in every fifth CIED patient. Too much slack in the lead significantly increases the risk of technical difficulties during the procedure. This is related to the presence of fibrous tissue on the leads and its role in the formation of adhesions, which can only be visualized via echocardiography. Long leads chronically adhere to the anatomical structures of the heart, and the touching of other leads cause lead-on-lead adhesion, thus becoming a major obstacle to safe lead dissection, freeing and extraction.

The displacement of the lead tip beyond the heart wall into the pericardial space may, apart from pacing disturbances, result in the presence of fluid in the pericardial sac or other pericardial reaction. Most often, this problem concerns ventricular leads implanted in the apical part of the right ventricle. Available evidence shows that perforation is a significant predictor of complications during the procedure. Hence, it is necessary to pay attention to the position of the ventricular lead tip during preoperative evaluation, especially when there is such a suspicion (pacing disturbances or a lead located outside the outline of the heart in fluoroscopy).

It is obvious, however, that when extracting leads with a shorter dwell time, the procedure is likely to be of low complexity, the risk of major complications is negligible and selected patients may even be discharged on the same day [39]. 

Lead-related valve dysfunction may be due to lead interference with tricuspid valve function. It has not been confirmed that the presence of LDTVD significantly influences the course of the procedure, except for when there is lead adhesion to the tricuspid apparatus. The presence of adhesions is associated with a significantly more frequent use of additional tools, which may result in damage to the leaflets or the tendon threads, as described in our previous studies [17]. The presence of LDTVD is associated with a worse long-term prognosis [40,41,42,43]. Therefore, a proper diagnosis and an appropriate management before progression of right heart dysfunction and significant stretching of the tricuspid ring are of major importance. A well-established treatment is the removal of the lead causing dysfunction and the implantation of a new one under the guidance of TEE, via the tricuspid valve or the use of alternative pacing sites (e.g., coronary sinus, His bundle). The width of the tricuspid ring is a predictor of improved tricuspid valve function after TLE, and therefore its measurement is an important part of the clinical assessment before qualification for lead extraction [44,45].

In summary, it should be noted that preoperative TEE is not only used to detect vegetations but also, and perhaps primarily, to assess the amount of fibrous lead encapsulation, which has a fundamental impact on TLE complexity and difficulty level and the risk of major complications. TEE performed immediately before TLE provides the operator with a lot of important information and helps them to be better prepared for the expected difficulties. Preoperative TEE, performed earlier and outside the operating room, may have an impact on the clinical decision-making process, including regarding transferring potentially more difficult patients to a more experienced center and having the procedure performed by the most experienced operator. However, an earlier TEE examination performed without general anesthesia may cause discomfort in the patient, and is never as accurate as an examination performed under general anesthesia. In a high-volume center it is not justified, but may be considered in selected patients in low-volume centers.

## 5. Conclusions

1. Performing a TEE before TLE provides the operator with a lot of important information. Apart from the visualization of possible vegetations, it can detect various forms of lead-related scar tissue.
2. Such findings as floating scar tissue attached to the lead, lead thickening, adhesion of leads to the anatomical structures of the heart, lead-to-lead binding, any form of scar tissue and the presence of abnormally long lead loops in the heart are associated with an increased complexity of the procedure and the risk of major complications.
3. The presence of vegetations has no influence on the level of procedure complexity or the risk of major complications, but significantly increases 1-year mortality.

## 6. Study Limitations

The present study is based on a single-center, observational, prospective experience. TLE was performed using only mechanical systems; laser energy was not applied to free the leads. We did not compare the diagnostic sensitivity of TEE and ICE.

## Figures and Tables

**Table 1:** Characteristics of the study groups.

	All Patients N = 1191	Non-Infectious A: N = 930	Local Pocket Infection B: N = 63	Systemic Infection C: N = 198
	Mean ± SD n (%)	Mean ± SD n (%)	Mean ± SD n (%)	Mean ± SD n (%)
		P (A vs. B) Chi^2^/“U” M-W Test	P (B vs. C) Chi^2^/“U” M-W Test	P (A vs. C) Chi^2^/ “U” M-W Test
Patient age at examination (years)	67.12 ± 14.74	66.07 ± 15.12 *p* = 0.004	71.60 ± 10.59 *p* = 0.584	70.63 ± 12.71 *p* < 0.001
Patient age at first CIED implantation (years)	57.06 ± 16.25	55.44 ± 17.22 *p* = 0.006	61.56 ± 10.53 *p* = 0.378	63.19 ± 13.40 *p* < 0.001
Female	464 (38.96)	394 (42.37) *p* = 0.179	21 (33.33) *p* = 0.239	49 (24.75) *p* < 0.001
Ischemic heart disease	767 (64.40)	581 (62.47) *p* = 0.001	53 (84.13) *p* = 0.015	133 (67.17) *p* = 0.244
Cardiomyopathy	160 (13.43)	115 (12.37) *p* = 0.399	5 (7.94) *p* = 0.040	40 (20.20) *p* = 0.005
Valvular heart disease	46 (3.86)	36 (3.87) *p* = 0.560	1 (1.59) *p* = 0.491	9 (4.55) *p* = 0.810
Other etiology (post inflammatory, congenital, unknown, surgical, post-ablation)	218 (18.30)	198 (21.29) *p* = 0.026	4 (6.35) *p* = 0.859	16 (8.08) *p* < 0.001
Permanent atrial fibrillation	275 (29.09)	206 (22.15) *p* = 0.886	14 (22.22) *p* = 0.859	55 (27.78) *p* = 0.107
Congestive heart failure	337 (28.30)	249 (26.77) *p* = 0.927	16 (25.40) *p* = 0.147	72 (36.36) *p* = 0.009
NYHA class III–IV	208 (17.36)	142 (15.27) *p* = 0.712	8 (12.70) *p* = 0.013	58 (29.29) *p* < 0.001
Diabetes (any)	251 (21.08)	176 (18.95) *p* = 0.351	11 (17.46) *p* = 0.035	64 (32.32) *p* = 0.014
Renal failure (any)	279 (23.43)	190 (20.43) *p* = 0.858	14 (22.22) *p* = 0.033	75 (37.88) *p* < 0.001
Charlson co-morbidity index (points)	4.97 ± 3.78	4.66 ± 3.72 *p* = 0.245	5.22 ± 3.30 *p* = 0.040	6.35 ± 3.92 *p* < 0.001
LVEF [%]	48.38 ± 15.52	49.22 ± 15.33 *p* = 0.984	49.18 ± 14.28 *p* = 0.029	44.18 ± 16.16 *p* < 0.001
LVEDD [mm]	54.20 ± 9.79	53.67 ± 9.56 *p* = 0.711	54.13 ± 9.37 *p* = 0.079	56.76 ± 10.60 *p* < 0.001
RVEDD [mm]	31.54 ± 3.33	31.32 ± 5.28 *p* = 0.639	31.00 ± 4.52 *p* = 0.170	32.07 ± 5.61 *p* = 0.077
SPAP [mmHg]	30.44 ± 11.99	29.54 ± 4.25 *p* = 0.002	31.65 ± 11.96 *p* = 0.152	34.31 ± 13.05 *p* < 0.001
Mitral regurgitation III–IV grade	192 (16.12)	141 (15.16) *p* = 0.995	9 (14.29) *p* = 0.305	42 (21.21) *p* = 0.047
Tricuspid regurgitation III–IV grade	286 (24.01)	214 (23.01) *p* = 0.153	20 (31.75) *p* = 0.493	52 (26.26) *p* = 0.375
Pacemaker (any)	833 (81.78)	683 (73.44) *p* = 0.948	44 (69.84) *p* = 0.033	106 (53.54) *p* < 0.001
ICD (any)	256 (21.94)	170 (18.28) *p* = 0.356	15 (23.81) *p* = 0.705	54 (27.27) *p* = 0.005
CRT-D	102 (8.56)	60 (6.45) *p* = 0.816	4 (6.35) *p* = 0.027	38 (19.19) *p* < 0.001

CIED—cardiac implantable electronic device, NYHA—New York Heart Association (functional class), LVEF—left ventricular ejection fraction, RVEDD—right ventricular end diastolic diameter, SPAP—systolic pulmonary artery pressure, CRT-D—cardiac resynchronization therapy cardioverter defibrillator.

**Table 2:** Echocardiographic findings before TLE according to indications for lead removal.

	Comparison of Patient Groups Divided According to Indications for TLE			
Variable	All Patients N = 1191	Non-Infectious A: N = 930	Local Pocket Infection B: N = 63	Systemic Infection C: N = 198
	Mean ± SD n (%)	Mean ± SD n (%)	Mean ± SD n (%)	Mean ± SD n (%)
		P (A vs. B) Chi^2^/M-W U test	P (B vs. C) Chi^2^/M-W U test	P (A vs. C) Chi^2^/M-W U test
Lead dependent tricuspid valve dysfunction reference diagnosis (LDTVD)	60 (5.31)	57 (6.53) *p* = 0.494	2 (3.18) *p* = 0.292	1 (0.51) *p* = 0.002
LDTVD confirmed	80 (6.72)	71 (7.63) *p* = 0.494	4 (6.35) *p* = 0.865	5 (2.52) *p* = 0.065
Prop of the leaflet by the lead	26 (2.18)	22 (2.37) *p* = 0.985	2 (3.18) *p* = 0.529	2 (1.01) *p* = 0.353
Pulling down the leaflet by the lead (immobilization)	49 (4.11)	43 (4.62) *p* = 0.796	3 (4.76) *p* = 0.310	3 (1.52) *p* = 0.070
Lead impingement of the valve or presence (irritation)	3 (0.25)	3 (0.32) *p* = 0.463	0 (0.00) N	0 (0.00) *p* = 0.698
Perforation of the leaflet by the lead	1 (0.08)	1 (0.11) *p* = 0.823	0 (0.00) N	0 (0.00) *p* = 0.936
Lead adhesion to the leaflet	1 (0.08)	1 (0.11) *p* = 0.823	0 (0.00) N	0 (0.00) *p* = 0.936
Vegetation-like masses	41 (3.44)	11 (1.18) *p* = 0.806	0 (0.00) *p* = 0.002	30 (15.15) *p* = 0.001
Scar tissue surrounding the lead	212 (17.80)	190 (20.43) *p* = 0.034	3 (4.76) *p* = 0.336	19 (9.60) *p* < 0.006
Lead thickening	435 (36.52)	335 (36.02) *p* = 0.845	24 (38.10) *p* = 0.914	76 (38.38) *p* = 0.585
Lead adhesion to the heart structures (any)	338 (29.34)	293 (32.77) *p* = 0.161	14 (22.22) *p* = 0.312	31 (15.90) *p* < 0.001
Lead adhesion to the SVC	108 (9.07)	96 (10.32) *p* = 0.990	6 (9.52) *p* = 0.072	6 (3.03) *p* = 0.002
Lead adhesion to the RA wall	116 (9.74)	100 (10.75) *p* = 0.924	6 (9.52) *p* = 0.323	10 (5.05) *p* = 0.020
Lead adhesion to the tricuspid apparatus	133 (11.17)	121 (13.01) *p* = 0.036	2 (3.18) *p* = 0.784	10 (5.05) *p* = 0.002
Lead adhesion to the RV wall	167 (14.02)	144 (15.48) *p* = 0.273	6 (9.52) *p* = 0.979	17 (8.59) *p* = 0.016
Lead-on-lead adhesion	352 (29.56)	306 (32.90) *p* = 0.106	14 (22.22) *p* = 0.363	32 (16.16) *p* < 0.001
No scar tissue (as defined above)	490 (41.14)	360 (38.71) *p* = 0.044	33 (52.38) *p* = 0.899	97 (48.99) *p* = 0.002
One form of scar tissue (as defined above)	267 (22.42)	197 (21.18) *p* = 0.808	12 (19.05) *p* = 0.151	58 (29.29) *p* = 0.017
Two or more (multiple) forms of scar tissue (as defined above)	434 (36.44)	373 (40.11) *p* = 0.093	18 (28.75) *p* = 0.043	43 (21.72) *p* < 0.001
Any form of scar tissue in a single patient [number]	2.17 ± 1.26	2.26 ± 1.29 *p* = 0.169	2.03 ± 1.16 *p* = 0.019	1.68 ± 0.98 *p* < 0.001
Blood clots on the lead	108 (9.07)	102 (10.97) *p* = 0.032	1 (1.59) *p* = 0.960	5 (2.53) *p* < 0.001
Vegetations visible in TTE	55 (4.63)	0 (0.00) N	0 (0.00) *p* < 0.001	55 (28.06) *p* < 0.001
Vegetations visible in TEE	154 (12.93)	0 (0.00) N	0 (0.00) *p* < 0.001	154 (77.78) *p* < 0.001
Vegetation diameter > 2 cm	32 (2.69)	0 (0.00) N	0 (0.00) *p* < 0.001	32 (16.16) *p* < 0.001
Multiple vegetations	111 (9.32)	0 (0.00) N	0 (0.00) *p* < 0.001	111 (56.06) *p* < 0.001
Lead vegetation (attached)	149 (98.03)	0 (0.00) N	0 (0.00) *p* < 0.001	149 (98.03) *p* < 0.001
Lead loops in the heart (any)	231 (19.40)	179 (19.25) *p* = 0.900	12 (19.05) *p* = 0.985	40 (20.20) *p* = 0.834
Lead loop in the RA	173 (14.58)	133 (14.33) *p* = 0.855	9 (14.52) *p* = 0.950	31 (15.74) *p* = 0.704
Leas loop in the TV	40 (3.36)	31 (3.34) *p* = 0.696	1 (1.59) *p* = 0.594	8 (4.06) *p* = 0.779
Lead loop in the RV or PA	45 (3.78)	37 (3.98) *p* = 0.980	3 (4.76) *p* = 0.633	5 (2.54) *p* = 0.439
Lead perforations and pericardial fluid				
Sure perforation	121 (10.16)	110 (11.83) *p* = 0.058	2 (3.18) *p* = 0.859	9 (4.55) *p* = 0.006
Penetration	66 (5.54)	54 (5.81) *p* = 0.921	4 (6.35) *p* = 0.733	8 (4.04) *p* = 0.492
Invisible tip of the lead in cases with suspicion of perforation	16 (1.34)	15 (1.61) *p* = 0.630	0 (0.00) *p* = 0.563	1 (0.51) *p* = 0.419
Fluid in epicardial space due to lead perforation				
Epicardial fluid independent of perforation; n (%)	46 (3.86)	33 (3.56) *p* = 0.247	0 (0.00) *p* = 0.071	13 (6.57) *p* = 0.083
Residual/very thin layer of fluid independent of perforation	2 (0.17)	1 (0.11) *p* = 0.279	1 (1.59) *p* = 0.545	0 (0.00) *p* = 0.394
Epicardial fluid related to perforation	30 (2.52)	22 (2.37) *p* = 0.972	1 (1.59) *p* = 0.702	7 (3.53) *p* = 0.493
Residual/very thin layer of fluid related to perforation	16 (1.34)	15 (1.61) *p* = 0.630	0 (0.00) *p* = 0.585	1 (0.51) *p* = 0.387

LDTVD—lead dependent tricuspid valve dysfunction, SVC—superior vena cava, RA—right atrium, RV—right ventricle, TLE—transvenous lead extraction, TTE—transthoracic echocardiography, PA—pulmonary artery.

**Table 3:** TEE findings for prediction of TLE complexity, major complications and 1-year mortality after TLE.

Echocardiographic Predictors of Complex TLE								
	**Complex TLE**		**Univariable Regressio**n			**Multivariable Regression**		
	**Yes** **N = 287**	**No** **N = 904**	**OR**	**95%CI**	***p***	**OR**	**95%CI**	*p*
	**Chi^2^ Test**							
Vegetation-like masses	5 (1.74)	36 (3.98) *p* = 0.104	0.889	0.420–1.881	0.757			
Scar tissue surrounding the lead	62 (21.60)	150 (16.59) *p* = 0.065	1.313	0.937–1.841	0.113			
Lead thickening	135 (47.30)	300 (33.19) *p* < 0.001	2.186	1.665–2.871	*p* < 0.001	1.536	1.123–2.102	0.007
Lead adhesion to the heart structures (any)	130 (47.27)	208 (23.72) *p* < 0.001	1.564	1.421–1.722	*p* < 0.001	2.531	1.821–3.518	*p* < 0.001
Lead-on-lead adhesion	124 (43.20)	228 (25.22) *p* < 0.001	2.151	1.595–2.902	*p* < 0.001	1.216	0.848–1.743	0.287
Any form of scar tissue	213 (74.22)	488 (53.98) *p* < 0.001	1.573	1.174–2.108	0.002			
Blood clots on the lead	21 (7.32)	87 (9.62) *p* = 0.286	0.761	0.458–1.265	0.291			
Vegetations visible in TEE	24 (8.36)	130 (14.38) *p* = 0.011	1.235	0.669–2.277	0.499	1.368	0.723–2.589	0.334
LDTVD confirmed	19 (6.62)	61 (6.75) *p* = 0.952	1.179	0.702–1.978	0.533			
Lead loops in the heart (any)	78 (27.18)	153 (16.93) *p* < 0.001	2.132	1.559–2.916	*p* < 0.001	1.632	1.152–2.312	0.006
Sure perforation	28 (9.76)	93 (10.28) *p* = 0.883	0.789	0.498–1.249	0.311			
Penetration	10 (3.48)	56 (6.19) *p* = 0.110	0.924	0.593–1.439	0.727			
Epicardial fluid independent of perforation	9 (3.14)	37 (4.09) *p* = 0.577	0.817	0.403–1.657	0.575			
Epicardial fluid related to perforation	7 (2.44)	23 (2.54) *p* = 0.907	0.758	0.376–1.528	0.437			
Echocardiographic predictors of major complications during TLE								
	**Major complications**		**Univariable regression**			**Multivariable regression**		
	**Yes** **N = 29**	**No** **N = 1162**	**OR**	**95%CI**	***p***	**OR**	**95%CI**	***p***
	**Chi^2^ test**							
Vegetation-like masses	2 (6.90)	39 (3.36) *p* = 0.605	3.211	0.933–11.051	0.064	4.080	1.038–16.05	0.044
Scar tissue surrounding the lead	12 (41.38)	200 (17.21) *p* = 0.002	2.813	1.317–6.009	0.007	1.863	0.813–4.268	0.141
Lead thickening	22 (75.86)	413 (35.54) *p* < 0.001	4.939	2.178–11.20	*p* < 0.001	2.389	1.050–5.761	0.049
Lead adhesion to the heart structures (any)	23 (82.14)	315 (28.03) *p* < 0.001	2.171	1.759–2.680	*p* < 0.001	6.341	2.354–17.08	*p* < 0.001
Lead-on-lead adhesion	24 (82.75)	328 (28.23) *p* < 0.001	4.841	2.318–10.11	*p* < 0.001	2.083	0.921–4.711	0.078
Any form of scar tissue	27 (93.10)	674 (58.00) *p* < 0.001	5.165	1.558–17.13	0.007			
Blood clots on the lead	1 (3.44)	107 (9.21) *p* = 0.459	0.356	0.048–2.642	0.312			
Vegetations visible in TEE	2 (6.89)	152 (13.08) *p* = 0.484	0.445	0.105–1.887	0.272			
Other TEE findings before TLE								
LDTVD confirmed	1 (3.44)	79 (6.80) *p* = 0.737	0.477	0.064–3.553	0.469			
Lead loops in the heart (any)	5 (17.12)	226 (19.45) *p* = 0.953	1.303	0.551–3.078	0.546			
Sure perforation	1 (3.45)	120 (10.33) *p* = 0.357	0.279	0.038–2.066	0.211			
Penetration	3 (10.32)	63 (5.42) *p* = 0.463	1.273	0.438–3.704	0.657			
Epicardial fluid independent of perforation	0 (0.00)	46 (3.96) *p* = 0.545	0	0–0	0			
Epicardial fluid related to perforation	0 (0.00)	30 (2.58) *p* = 0.782	0	0–0	0			
Echocardiographic predictors of death during 1-year follow-up								
	**Death during 1-year follow-up**		**Univariable Cox regression**			**Multivariable Cox regression**		
	**Yes** **N = 96**	**No** **N = 1095**						
	**Chi^2^ test**							
Vegetation-like masses	3 (3.13)	38 (3.47) *p* = 0.909	0.836	0.265–2.641	0.761			
Scar tissue surrounding the lead	8 (8.33)	204 (18.63) *p* = 0.017	0.412	0.200–0.851	0.016	0.690	0.328–1.451	0.328
Lead thickening	26 (27.83)	409 (37.35) *p* = 0.058	0.664	0.423–1.042	0.075	0.642	0.396–1.039	0.071
Lead adhesion to the heart structures (any)	17 (17.71)	321 (30.40) *p* = 0.021	0.704	0.578–0.859	*p* < 0.001	0.582	0.321–1.056	0.075
Lead-on-lead adhesion	19 (19.79)	333 (30.41) *p* = 0.044	0.436	0.226–0.839	0.013	0.622	0.305–1.267	0.191
Any form of scar tissue	47 (48.96)	654 (59.73) *p* = 0.052	1.251	0.816–1.92	0.305			
Blood clots on the lead	6 (6.25)	102 (9.32) *p* = 0.414	0.708	0.310–1.618	0.413			
Vegetations visible in TEE	36 (37.50)	118 (10.78) *p* < 0.001	8.895	5.567–14.22	*p* < 0.001	7.254	4.475–11.76	*p* < 0.001
Other TEE findings before TLE								
LDTVD confirmed	8 (8.33)	72 (6.58) *p* = 0.655	1.311	0.636–2.703	0.464			
Lead loops in the heart (any)	11 (11.46)	220 (20.09) *p* = 0.055	0.581	0.317–1.063	0.078	0.747	0.399–1.398	0.361
Sure perforation	6 (6.32)	115 (10.50) *p* = 0.252	0.64	0.297–1.381	0.255			
Penetration	4 (4.17)	62 (5.66) *p* = 0.703	1.018	0.543–1.906	0.956			
Epicardial fluid independent of perforation	5 (5.21)	41 (3.74) *p* = 0.662	0.477	0.118–1.934	0.300			
Epicardial fluid related to perforation	1 (1.04)	29 (2.65) *p* = 0.533	1.624	0.711–3.71	0.250			

TEE—transesophageal echocardiography, LDTVD—lead dependent tricuspid valve dysfunction.

## Data Availability

Data are available upon request to authors.

## Supplementary Materials

The following supporting information can be downloaded at: https://www.mdpi.com/article/10.3390/jcm13175278/s1, Movie S1. TEE 2D Floating scar tissue connected to the lead; Movie S2. TEE 3D Blood cloth on the lead; Movie S3. TEE 2D Vegetation-like masses on the lead in rght atrium; Movie S4. TEE 2D Bacterial vegetation on the lead in the right atrium moving to the right ventricle; Movie S5. TEE 2D Thickening of the leads and inter-lead adhesion showing common mobility; Movie S6. TEE 2D Interlead adhesion and to the m-atrial septum as well as to the atrial wall. Narrowing of the vena cava superior is caused by the thickened leads and pathologic connective tissue; Movie S7. TEE 3D The lead in the tricuspid valve supports the septal leaflet and hinders proper coaptation of the leaflets; Movie S8.TEE 2D Perforation wall right ventricle through the lead to the pericardial; Movie S9. TEE 2D Penetration of the right ventricle wall with the lead, the tip of lead deeply at the interface between the muscle and the pericardium is referred; Movie S10. TEE 2D A long loop of left ventricular lead translocating to the pulmonary trunk.
